# Clinical significance of gefitinib antitumor activity in patients with lung adenocarcinoma

**DOI:** 10.3892/ol.2014.2664

**Published:** 2014-11-04

**Authors:** ZHUN WANG, QIAN-BO HAN, JIA-LEI GU, XIN-MIN YU, XIAO-JIANG SUN, QING-REN LIN, JUN FANG, YUE-ZHEN WANG, YA-PING XU, WEI-MIN MAO

**Affiliations:** 1 Department of Radiation Oncology, Zhejiang Cancer Hospital, Hangzhou, Zhejiang 310022, P.R. China; 2 Department of Medical Oncology, Zhejiang Cancer Hospital, Hangzhou, Zhejiang 310022, P.R. China; 3 Department of Thoracic Surgery, Zhejiang Cancer Hospital, Hangzhou, Zhejiang 310022, P.R. China

**Keywords:** non-small cell lung cancer, gefitinib, targeted therapy

## Abstract

Non-small cell lung cancer is a subtype of adenocarcinoma, which has previously shown positive responses to gefitinib. The aim of the current study was to determine a clinical profile of gefitinib-induced disease controls for patients with lung adenocarcinoma. Retrospective evaluation of the clinical characteristics of 52 lung adenocarcinoma patients, enrolled at the Zhejiang Cancer Hospital (Hangzhou, China) between October 2004 and August 2008, was undertaken. All patients received gefitinib (250 mg/day orally) until disease progression or until an unacceptable toxicity was observed. Of the 52 patients, complete response (CR) and partial response (PR) rates were 23.1% (12/52) and 57.7% (30/52), respectively. An additional 19.2% (10/52) of patients demonstrated stable disease (SD) after three months of treatment with gefitinib. Disease control was observed in the primary lesion, and tumor metastasis to the lungs, brain, adrenal glands, pleura, peritoneum, pericardium, bone and lymph nodes was identified. The one-year progression-free survival (PFS) and overall survival (OS) rates were 74.8 and 78.0%, respectively. Multivariate analysis revealed that female patients were associated with significantly longer survival times when compared with males (hazard ratio, 0.077; 95% confidence interval [CI], 0.007–0.083; P=0.035). One-year PFS and OS rates in CR, PR and SD patients were 77.8, 73.9 and 33.3%, and 89.2, 79.8 and 33.7%, respectively, although neither difference was identified to be statistically significant. In addition, the median OS of SD patients was 12 months (95% CI, 7.2–16.8 months). Brain metastasis was the major site of disease progression (23.1%). Gefitinib treatment for patients with lung adenocarcinoma showed a marked long-term survival benefit, even in SD patients. However, further studies are required to analyze the efficacy of gefitinib in penetrating the blood-brain barrier in order to prolong PFS in patients with lung adenocarcinoma.

## Introduction

Lung cancer is one of the leading causes of cancer-related mortalities worldwide (1). The incidence rate of lung cancer is increasing in Asia, particularly in China. However, despite chemo- and radiation therapy producing survival benefits in patients with advanced non-small cell lung cancer (NSCLC), the survival rate of lung cancer remains particularly low. Therefore, there is a clear requirement for novel and more effective control strategies for lung cancer. Thus, inhibition of epidermal growth factor receptor (EGFR) tyrosine kinase has emerged as a novel therapeutic option for the treatment of NSCLC. Gefitinib, an oral EGFR tyrosine kinase inhibitor (TKI), is a leading agent in this class of novel therapeutic agents. Two major phase II trials (2,3), large expanded access programs across the world (4–6), as well as other studies (7,8) have demonstrated a higher objective response rate and prolonged survival time in females and never-smoking adenocarcinoma patients of East-Asian origin. Furthermore, a prospective trial, which administered gefitinib as a first-line therapy for advanced lung adenocarcinoma patients that were never-smokers, was conducted in South Korea and was found to be highly efficacious (9).

Molecularly, NSCLC cells demonstrate mutation (10–12) and amplification (13,14) of the EGFR gene. Treatment with gefitinib has demonstrated that NSCLC patients with such mutations or amplifications, as well as expression of phosphorylated Akt (15) and ErbB3 (16) are associated with an improved outcome (17–23). However, it is often difficult to obtain the tumor tissue of patients to detect gene status. Therefore, it is necessary to investigate the correlation between the clinical features of NSCLC and the prognosis that is determined in the clinical setting. The current study was conducted to investigate such correlations in Chinese lung adenocarcinoma patients using gefitinib-induced disease controls.

## Patients and methods

### Patient population

A total of 52 patients were recruited between October 2004 and August 2008 at the Zhejiang Cancer Hospital (Hangzhou, China). The clinical characteristics of the patients are summarized in Table I. All patients were histologically or cytologically diagnosed with lung adenocarcinoma. The interval between the final cycle of chemotherapy and administration of the gefitinib treatment was ≥30 days. The present study was approved by the Institutional Review Board of Zhejiang Cancer Hospital and performed in accordance with the recommendations of the Declaration of Helsinki with regard to the biomedical research involving human subjects. Written informed consent was obtained from all patients.

### Treatment schedule

Gefitinib (ZD1839; AstraZeneca, Wilmington, DE, USA) was administered orally at a dosage of 250 mg/day until disease progression, an unacceptable type of toxicity or withdrawal of patient consent. No other chemotherapeutic agents were administered during the course of the study. Medications for symptomatic relief, such as analgesics and bisphosphonates were permitted. Twenty-three patients with brain metastases received whole brain radiotherapy (WBRT) during the gefitinib treatment period. Seventeen patients with symptomatic bone metastases received palliative radiotherapy. Baseline evaluations were performed within the week prior to enrollment, including a complete medical history and physical examination, laboratory tests (whole blood counts, and liver and renal function), electrocardiograms, thorax computed tomography (CT), ultrasonography of the abdomen, bone scintigraphy and brain CT or magnetic resonance imaging (MRI). Furthermore, blood counts, and liver and renal function tests were performed prior to each 30-day treatment cycle. Follow-up data after gefitinib treatment (for example, recurrence, metastasis, vitals status, mortality and cause of mortality) were obtained from the patient records.

### Response assessment and evaluation of toxicity

Response to the treatment was evaluated by CT, MRI and ultrasonography of the abdomen, as well as bone scintigraphy. The response rate was recorded according to the Response Evaluation Criteria In Solid Tumors (24). For data analysis, complete response (CR) and partial response (PR) were combined and termed responders. CR and PR refer to a sustained response over a period of four weeks or longer, however, stable disease (SD) refers to a response persisting for eight weeks or more. The type of toxicity was evaluated according to the National Cancer Institute Common Toxicity Criteria (25) and the worst scores obtained during treatment were recorded.

### Statistical analysis

Progression-free survival (PFS) and overall survival (OS) were measured from the date of initiation of gefitinib treatment until disease progression or mortality, respectively. The survival curves were calculated using the Kaplan-Meier method. Multivariate survival analysis was performed using a Cox proportional hazard regression model with a backward stepwise procedure. The considered variables included age, gender, cigarettes per year, Eastern Cooperative Oncology Group performance status (ECOG PS) score, type of metastatic lesion and whether gefitinib is the first-line treatment. Statistical analysis was performed using SPSS software, version 13.0 (SPSS Inc., Chicago, IL, USA). All probability values were two-tailed and P<0.05 was considered to indicate a statistically significant difference.

## Results

### Clinical significance of gefitinib treatment in patients with NSCLC

The clinical characteristics of 52 patients are summarized in Table I. Of the 52 patients who participated in the present study, CR and PR rates were 23.1% (12/52) and 57.7% (30/52), respectively. An additional 19.2% (10/52) of patients demonstrated SD.

Table II shows the response of primary and various metastatic lesions to gefitinib treatment. For example, the primary tumor size was reduced by 54.8±19.8% (mean ± standard deviation) in the five assessable patients. Prior to gefitinib administration, 18 patients exhibited intrapulmonary metastases, 17 of which regressed with gefitinib treatment. Intrapulmonary metastases in 11 patients were assessed as the target lesions and the extent of the reduction in size was 79.1±21.6%. Twenty-three patients exhibited brain metastases, 18 of which regressed as a result of gefitinib administration. The brain metastases in 21 patients were assessed as the target lesions and the shrinkage of these was 68.5±27.9%. The response of lymph node and liver metastases, and pleural effusion to gefitinib was also favorable. Bone metastases was initially detected in 27 patients and remained unchanged following gefitinib treatment, as identified by bone scintigraphy.

**Figure 1:**
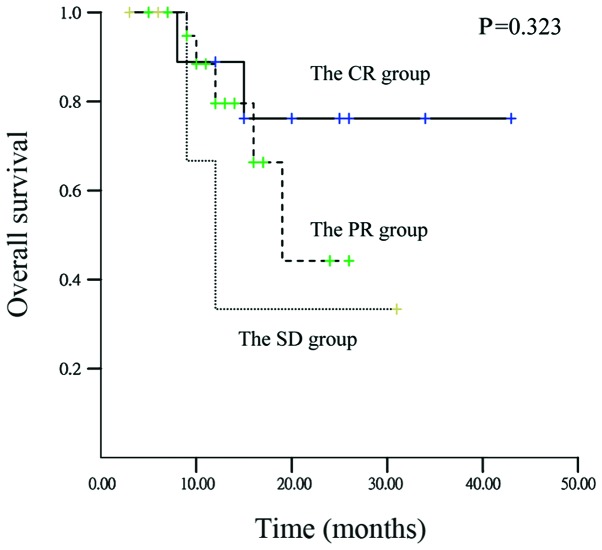
Kaplan-Meier survival curves of 52 patients with lung adenocarcinoma treated with gefitinib for three months. CR, complete response; PR, partial response; SD, stable disease. P<0.05 indicates a statistically significant difference.

One-year PFS and OS rates were 74.8 and 78.0%, respectively. Multivariate analysis revealed that female patients had significantly longer survival rates when compared with male patients. Other factors, such as age, smoking status, ECOG PS, metastatic lesions and gefitinib as first-line treatment, did not exhibit a significant association with longer survival time (Table III). One-year OS rates in CR, PR and SD patients were 89.2, 79.8 and 33.7%, respectively. One-year PFS rates in CR, PR, and SD patients were 77.8, 73.9 and 33.3%, respectively; however, there were no statistically significant differences detected in OS (P=0.323) and PFS (P=0.379) among patients with CR, PR and SD. Fig. 1 shows the OS curves of patients with CR, PR and SD. Median OS of the SD patients was 12 months (95% confidence interval [CI], 7.2–16.8 months).

### Toxicity and safety issues

Treatment with gefitinib was generally well tolerated. The most common types of toxicity were rashes (88%) and diarrhea (48%; Table IV). Grade II diarrhea was well controlled by supportive care and grade III diarrhea occurred in six patients (12%). Two patients suffered from hand-foot syndrome. However, none of the patients refused continuous treatment with gefitinib.

### Disease progression following gefitinib-induced disease control

At the time of data analysis, with a median follow-up time of 21 months, a total of 25 (48.1%) patients exhibited disease progression and their treatment was discontinued. The sites of initial disease progression following gefitinib-induced disease control among the 52 patients are summarized in Table V. The disease progression sites included the primary lesion (3/52; 5.8%), the intrapulmonary (5/52; 9.6%), brain (12/52; 23.1%) and bone (7/52; 13.5%) lesions, pleural effusion (5/52; 9.6%), and the peritoneum (2/52; 3.8%) and liver lesions (2/52; 3.8%).

## Discussion

In the present retrospective study, the response and disease progression of primary and metastatic lesions was analyzed in lung adenocarcinoma patients who achieved PR, CR or SD following three months of treatment with gefitinib. A more positive outcome was observed in the present study compared with previous studies (21,26,27). The CR and PR rates were 23.1% (12/52) and 57.7% (30/52), respectively. An additional 19.2% (10/52) of patients achieved SD. One-year PFS and OS rates were 74.8 and 78.0%, respectively. Multivariate analysis showed that female patients had significantly longer survival times when compared with male patients. Fukuoka *et al* (2) reported that the PR and SD rates were 18.5 and 35.9%, respectively, with gefitinib administered at a dosage of 250 mg/day. In patients with either CR or PR, the median OS was reported as 13.3 months for the 250-mg/day group and 10.6 months for the 500-mg/day group. Mok *et al* (28) demonstrated that gefitinib was superior to chemotherapy as an initial treatment modality for lung adenocarcinoma among non-smokers or former light smokers in East Asia, despite the one-year PFS rate of 24.9%. Therefore, gefitinib treatment for patients with lung adenocarcinoma resulted in a marked survival benefit.

Previous studies have demonstrated that gefitinib produced a higher objective response rate and prolonged survival time in females and never-smoking adenocarcinoma patients of East Asian origin (7,8,29). In the current study, multivariate analysis revealed that female patients had a statistically significant association with longer survival time when compared with male patients, whereas other patient parameters, such as age, smoking status, ECOG PS, tumor metastasis or gefitinib as a first-line treatment were not associated with prolonged survival. The results from the current study were similar to previous reports (7,8). It is hypothesized that female patients demonstrate an improved response to gefitinib as a results of EGFR mutations, which occur more frequently in females (30).

The current study demonstrated that one-year PFS and OS rates in CR, PR and SD patients were 77.8, 73.9, 33.3%, and 89.2, 79.8 and 33.7%, respectively, although neither difference was identified to be statistically significant. However, a previous study has indicated that patients obtaining SD following gefitinib treatment had significantly longer OS than those with progressive disease (31). In addition, Yang *et al* (32) reported that the PFS times in dramatic, gradual, and local progression groups, following gefitinib treatment, were 9.3, 12.9 and 9.2 months, respectively (P=0.007). Furthermore, the OS for these groups was 17.1, 39.4, and 23.1 months, respectively (P<0.001). TKI continuation was identified to be superior to switching the type of chemotherapy in a subsequent setting for gradual progression (39.4 months vs. 17.8 months; P=0.02) (32). The above-mentioned findings indicate that patients achieving SD or gradual progression following gefitinib treatment may achieve long-term survival.

In the current study, brain metastases (23.1%) was the major site of disease progression after treatment with gefitinib. Similarly, Omuro *et al* (33) reported that the central nervous system (CNS) was the most frequent site of disease progression in patients with NSCLC after an initial response to gefitinib. This may be due to the presence of the intact blood-brain barrier, which gefitinib could not penetrate despite its low molecular weight. Fukuhara *et al* (34) reported that the concentration of gefitinib in the patient’s cerebrospinal fluid (CSF; 0.9 nM) was <1% of the serum concentration (117 and 132 nM, prior to and 2 h following drug re-administration, respectively) when treated with 250 mg/day gefitinib. In another study, Jackman *et al* (35)reported that increasing doses of gefitinib resulted in increasing concentrations of gefitinib in the CSF, with the concentration of gefitinib in the patients’ CSF varying from 6.2 to 18 nM, following a 500-mg dose, and reaching 42 nM following a 1,000-mg dose. Following administration of that regimen, the patients’ carcinomatous meningitis was controlled for ~four months (35). Conversely, our previous phase II study demonstrated that a concomitant treatment with gefitinib and WBRT in patients with brain metastases from NSCLC resulted in a favorable prognosis (36). Thus, further molecular studies are required to investigate the efficacy of gefitinib in penetrating the blood-brain barrier. The approach was previously investigated in China by those that conducted the ZhejiangCH06 trial, (NCT01158170) (37).

In conclusion, lung adenocarcinoma patients treated with gefitinib-induced disease control showed marked survival benefits. Furthermore, patients achieving SD with gefitinib treatment may achieve long-term survival. Further studies are required to analyze the efficacy of gefitinib in penetrating the blood-brain barrier.

## Figures and Tables

**Table I:** Characteristics of 52 patients (median age, 65 years; range, 34–84 years) with lung adenocarcinoma.

Characteristic	Patients, n	%
Age, years		
<65	25	48.1
≥65	27	51.9
Gender		
Male	19	36.5
Female	33	63.5
Cigarettes per year		
≥400	16	30.8
<400	4	7.7
Never-smoker	32	61.5
ECOG PS		
0–1	20	38.5
≥2	32	61.5
Therapy		
No previous chemotherapy regimens received	10	19.2
1 previous chemotherapy regimen	28	53.8
≥2 previous chemotherapy regimens	9	17.3
Radiation therapy	5	9.6

ECOG PS, Eastern Cooperative Oncology Group performance status.

**Table II:** Response of primary and metastatic lesions to gefitinib in the responders (partial and complete response groups combined).

Site	Patients, n	Improved patients, n	Patients with a target lesion, n	Tumor shrinkage of the target lesion, % (mean ± standard deviation)
Primary lesion	5	5	5	54.8±19.8
Metastastic lesions				
Intrapulmonary	18	17	11	79.1±21.6
Brain	23	18	21	68.5±27.9
Lymph nodes	12	10	12	47.5±10.5
Bone	27	0		
Pleura	13	12		
Other	6	5	3	41.7±9.7

**Table III:** Factors associated with overall survival according to multivariate analysis.

Parameter	Hazard ratio	95% CI	P-value
Age (<65 vs. ≥65 years)	0.000	0.000–7.823	0.978
Gender (Male vs. Female)	0.077	0.007–0.830	0.035
Cigarettes per year (<400 vs. ≥400)	8.238	0.763–88.984	0.082
Gefitinib as a first-line therapy (Yes vs. No)	0.815	0.102–6.519	0.847
Metastatic lesions (Intrapulmonary vs. brain vs. lymph nodes vs. bone vs. pleura vs. other)	0.392	0.041–3.795	0.419
ECOG PS (0–1 vs.≥2)	4.970	0.682–36.216	0.114

ECOG PS, Eastern Cooperative Oncology Group performance status; CI, confidence interval.

**Table IV:** Toxicity profile of the 52 patients following treatment with gefitinib.

	% (number of patients)				
	
Complication	Grade 0	Grade I	Grade II	Grade III	Grades I+II+III
Rash	12 (6)	19 (10)	65 (34)	4 (2)	88 (46)
Diarrhea	52 (27)	19 (10)	17 (9)	12 (6)	48 (25)
Mucositis	72 (37)	13 (7)	15 (8)	0 (0)	28 (15)
Liver dysfunction	87 (45)	12 (6)	2 (1)	0 (0)	13 (7)
Neutropenia	92 (48)	8 (4)	0 (0)	0 (0)	8 (4)
Lung toxicity	100 (52)	0 (0)	0 (0)	0 (0)	0 (0)
Hand-foot syndrome	96 (50)	4 (2)	0 (0)	0 (0)	0 (0)

**Table V:** Sites of initial disease progression following gefitinib-induced disease control.

Site	Patients, n	%
Primary lesion	3	5.8
Metastatic lesion		
Intrapulmonary	5	9.6
Brain	12	23.1
Bone	7	13.5
Pleura	5	9.6
Peritoneum	2	3.8
Liver	2	3.8

## References

[1] Jemal A, Siegel R, Ward E (2009). Cancer statistics, 2009. CA Cancer J Clin.

[2] Fukuoka M, Yano S, Giaccone G (2003). Multi-institutional randomized phase II trial of gefitinib for previously treated patients with advanced non-small-cell lung cancer (The IDEAL 1 Trial) [corrected]. J Clin Oncol.

[3] Kris MG, Natale RB, Herbst RS (2003). Efficacy of gefitinib, an inhibitor of the epidermal growth factor receptor tyrosine kinase, in symptomatic patients with non-small cell lung cancer: a randomized trial. JAMA.

[4] Jänne PA, Gurubhagavatula S, Yeap BY (2004). Outcomes of patients with advanced non-small cell lung cancer treated with gefitinib (ZD1839, ‘Iressa’) on an expanded access study. Lung Cancer.

[5] Santoro A, Cavina R, Latteri F (2004). Activity of a specific inhibitor, gefitinib (Iressa, ZD1839), of epidermal growth factor receptor in refractory non-small-cell lung cancer. Ann Oncol.

[6] Park J, Park BB, Kim JY (2004). Gefitinib (ZD1839) monotherapy as a salvage regimen for previously treated advanced non-small cell lung cancer. Clin Cancer Res.

[7] Konishi J, Yamazaki K, Kinoshita I (2005). Analysis of the response and toxicity to gefitinib of non-small cell lung cancer. Anticancer Res.

[8] Simon GR, Ruckdeschel JC, Williams C (2003). Gefitinib (ZD1839) in previously treated advanced non-small-cell lung cancer: experience from a single institution. Cancer Control.

[9] Lee DH, Han JY, Lee HG (2005). Gefitinib as a first-line therapy of advanced or metastatic adenocarcinoma of the lung in never-smokers. Clin Cancer Res.

[10] Lynch TJ, Bell DW, Sordella R (2004). Activating mutations in the epidermal growth factor receptor underlying responsiveness of non-small-cell lung cancer to gefitinib. N Engl J Med.

[11] Paez JG, Jänne PA, Lee JC (2004). EGFR mutations in lung cancer: correlation with clinical response to gefitinib therapy. Science.

[12] Okamoto I, Takahashi T, Okamoto H (2011). Single-agent gefitinib with concurrent radiotherapy for locally advanced non-small cell lung cancer harboring mutations of the epidermal growth factor receptor. Lung Cancer.

[13] Cappuzzo F, Hirsch FR, Rossi E (2005). Epidermal growth factor receptor gene and protein and gefitinib sensitivity in non-small-cell lung cancer. J Natl Cancer Inst.

[14] Hirsch FR, Herbst RS, Olsen C (2008). Increased EGFR gene copy number detected by fluorescent in situ hybridization predicts outcome in non-small-cell lung cancer patients treated with cetuximab and chemotherapy. J Clin Oncol.

[15] Cappuzzo F, Magrini E, Ceresoli GL (2004). Akt phosphorylation and gefitinib efficacy in patients with advanced non-small-cell lung cancer. J Natl Cancer Inst.

[16] Engelman JA, Jänne PA, Mermel C (2005). ErbB-3 mediates phosphoinositide 3-kinase activity in gefitinib-sensitive non-small cell lung cancer cell lines. Proc Natl Acad Sci USA.

[17] Mitsudomi T, Kosaka T, Endoh H (2005). Mutations of the epidermal growth factor receptor gene predict prolonged survival after gefitinib treatment in patients with non-small-cell lung cancer with postoperative recurrence. J Clin Oncol.

[18] Han SW, Kim TY, Hwang PG (2005). Predictive and prognostic impact of epidermal growth factor receptor mutation in non-small-cell lung cancer patients treated with gefitinib. J Clin Oncol.

[19] Hirsch FR, Varella-Garcia M, McCoy J (2005). Southwest Oncology Group: Increased epidermal growth factor receptor gene copy number detected by fluorescence in situ hybridization associates with increased sensitivity to gefitinib in patients with bronchioloalveolar carcinoma subtypes: a Southwest Oncology Group Study. J Clin Oncol.

[20] Tokumo M, Toyooka S, Kiura K (2005). The relationship between epidermal growth factor receptor mutations and clinicopathologic features in non-small cell lung cancers. Clin Cancer Res.

[21] Kim KS, Jeong JY, Kim YC (2005). Predictors of the response to gefitinib in refractory non-small cell lung cancer. Clin Cancer Res.

[22] Takano T, Ohe Y, Sakamoto H (2005). Epidermal growth factor receptor gene mutations and increased copy numbers predict gefitinib sensitivity in patients with recurrent non-small-cell lung cancer. J Clin Oncol.

[23] Riely GJ, Pao W, Pham D (2006). Clinical course of patients with non-small cell lung cancer and epidermal growth factor receptor exon 19 and exon 21 mutations treated with gefitinib or erlotinib. Clin Cancer Res.

[24] Eisenhauer EA, Therasse P, Bogaerts J (2009). New response evaluation criteria in solid tumors: revised RECIST guideline (version 1.1). Eur J Cancer.

[25] Trotti A, Byhardt R, Stetz J (2000). Common toxicity criteria: version 2.0. an improved reference for grading the acute effects of cancer treatment: impact on radiotherapy. Int J Radiat Oncol Biol Phys.

[26] Cella D, Herbst RS, Lynch TJ (2005). Clinically meaningful improvement in symptoms and quality of life for patients with non-small-cell lung cancer receiving gefitinib in a randomized controlled trial. J Clin Oncol.

[27] Yokouchi H, Yamazaki K, Kinoshita I (2007). Clinical benefit of readministration of gefitinib for initial gefitinib-responders with non-small cell lung cancer. BMC Cancer.

[28] Mok TS, Wu YL, Thongprasert S (2009). Gefitinib or carboplatin-paclitaxel in pulmonary adenocarcinoma. N Engl J Med.

[29] Park K, Goto K (2006). A review of the benefit-risk profile of gefitinib in Asian patients with advanced non-small-cell lung cancer. Curr Med Res Opin.

[30] Inoue A, Suzuki T, Fukuhara T (2006). Prospective phase II study of gefitinib for chemotherapy-naive patients with advanced non-small-cell lung cancer with epidermal growth factor receptor gene mutations. J Clin Oncol.

[31] Hotta K, Matsuo K, Ueoka H (2005). Continued gefitinib treatment after disease stabilisation prolongs survival of Japanese patients with non-small-cell lung cancer: Okayama Lung Cancer Study Group experience. Ann Oncol.

[32] Yang JJ, Chen HJ, Yan HH (2013). Clinical modes of EGFR tyrosine kinase inhibitor failure and subsequent management in advanced non-small cell lung cancer. Lung Cancer.

[33] Omuro AM, Kris MG, Miller VA (2005). High incidence of disease recurrence in the brain and leptomeninges in patients with nonsmall cell lung carcinoma after response to gefitinib. Cancer.

[34] Fukuhara T, Saijo Y, Sakakibara T (2008). Successful treatment of carcinomatous meningitis with gefitinib in a patient with lung adenocarcinoma harboring a mutated EGF receptor gene. Tohoku J Exp Med.

[35] Jackman DM, Holmes AJ, Lindeman N (2006). Response and resistance in a non-small-cell lung cancer patient with an epidermal growth factor receptor mutation and leptomeningeal metastases treated with high-dose gefitinib. J Clin Oncol.

[36] Ma S, Xu Y, Deng Q, Yu X (2009). Treatment of brain metastasis from non-small cell lung cancer with whole brain radiotherapy and Gefitinib in a Chinese population. Lung Cancer.

[37] Ma S, Xu Y (2010). Prophylactic Cranial Irradiation (PCI) in Erlotinib/Gefitinib-Responders With Non-small Cell Lung Cancer (NSCLC) (RT1001). ClinicalTrialsgov Identifier: NCT01158170.

